# Loss of Catecholaminergic Neuromodulation of Persistent Forms of Hippocampal Synaptic Plasticity with Increasing Age

**DOI:** 10.3389/fnsyn.2016.00030

**Published:** 2016-09-26

**Authors:** Hannah Twarkowski, Denise Manahan-Vaughan

**Affiliations:** ^1^Department of Neurophysiology, Medical Faculty, Ruhr University BochumBochum, Germany; ^2^International Graduate School of Neuroscience, Ruhr University BochumBochum, Germany

**Keywords:** dentate gyrus, dopamine D1/D5, noradrenaline, beta-adrenergic, LTP, synaptic plasticity, *in vivo*, rat

## Abstract

Neuromodulation by means of the catecholaminergic system is a key component of motivation-driven learning and behaviorally modulated hippocampal synaptic plasticity. In particular, dopamine acting on D1/D5 receptors and noradrenaline acting on beta-adrenergic receptors exert a very potent regulation of forms of hippocampal synaptic plasticity that last for very long-periods of time (>24 h), and occur in conjunction with novel spatial learning. Antagonism of these receptors not only prevents long-term potentiation (LTP) and long-term depression (LTD), but prevents the memory of the spatial event that, under normal circumstances, leads to the perpetuation of these plasticity forms. Spatial learning behavior that normally comes easily to rats, such as object-place learning and spatial reference learning, becomes increasingly impaired with aging. Middle-aged animals display aging-related deficits of specific, but not all, components of spatial learning, and one possibility is that this initial manifestation of decrements in learning ability that become apparent in middle-age relate to changes in motivation, attention and/or the regulation by neuromodulatory systems of these behavioral states. Here, we compared the regulation by dopaminergic D1/D5 and beta-adrenergic receptors of persistent LTP in young (2–4 month old) and middle-aged (8–14 month old) rats. We observed in young rats, that weak potentiation that typically lasts for *ca.* 2 h could be strengthened into persistent (>24 h) LTP by pharmacological activation of either D1/D5 or beta-adrenergic receptors. By contrast, no such facilitation occurred in middle-aged rats. This difference was not related to an ostensible learning deficit: a facilitation of weak potentiation into LTP by spatial learning was possible both in young and middle-aged rats. It was also not directly linked to deficits in LTP: strong afferent stimulation resulted in equivalent LTP in both age groups. We postulate that this change in catecholaminergic control of synaptic plasticity that emerges with aging, does not relate to a learning deficit *per se*, rather it derives from an increase in behavioral thresholds for novelty and motivation that emerge with increasing age that impact, in turn, on learning efficacy.

## Introduction

The catecholaminergic system, and particularly dopamine and noradrenaline, are substantially involved in state-dependent information processing in the central nervous system. The ventral tegmental area and the locus coeruleus are the major sources of hippocampal dopamine and noradrenaline, respectively. Both structures increase their firing rates when an animal is presented with novel stimuli (Aston-Jones and Bloom, 1981; Horvitz et al., 1997; Kitchigina et al., 1997) and dopamine and noradrenaline levels in the hippocampus increase during exploration of a novel environment (Ihalainen et al., 1999). In addition, the dopaminergic system is involved in reward-learning behavior (Schultz et al., 1993; Ungless, 2004).

In the hippocampus, activation of either dopamine D1/D5 receptors or beta-adrenergic receptors reduces the threshold for induction of synaptic plasticity that lasts for very long (>24 h) periods of time in rodents (Hansen and Manahan-Vaughan, 2014; Hagena et al., 2016). Correspondingly, weak synaptic potentiation (short-term potentiation, STP) is prolonged into persistent long-term potentiation (LTP) if patterned afferent stimulation occurs in the presence of dopamine D1/D5 receptor or beta-adrenergic receptor agonists (Kusuki et al., 1997; Li et al., 2003; Almaguer-Melian et al., 2005; Lemon and Manahan-Vaughan, 2006; Tenorio et al., 2010; Hagena and Manahan-Vaughan, 2012; Hansen and Manahan-Vaughan, 2015b). Moreover, hippocampal LTP is impaired by dopamine D1/D5 receptor or beta-adrenergic receptor antagonists (Stanton and Sarvey, 1985; Straube and Frey, 2003; Hansen and Manahan-Vaughan, 2015b). LTP is tightly associated with spatial learning and exploration of a novel environment results in the strengthening of STP into persistent (>24 h) LTP in rodents *in vivo* (Kemp and Manahan-Vaughan, 2004, 2008a; Hagena and Manahan-Vaughan, 2012). This facilitation of LTP through novel learning requires activation of both dopamine D1/D5 and beta-adrenergic receptors (Straube et al., 2003; Lemon and Manahan-Vaughan, 2006; Kemp and Manahan-Vaughan, 2008b; Hansen and Manahan-Vaughan, 2015a, b).

The catecholaminergic system may change with age (Popova and Petkov, 1989; Araki et al., 1997; Suzuki et al., 2001). But aging, despite the manifestation of attrition in cognition, including memory deficits (Perlmutter et al., 1981; Larrabee and McEntee, 1995), is not always a process of degeneration. Decline in cognitive ability with aging is related to changes in the hippocampus (Driscoll et al., 2003). These cognitive changes begin surprisingly early in mature adulthood in healthy humans (Ballesteros et al., 2013). We have observed deficits in object-place learning and in extinction learning that emerge in healthy middle-age in rats (Wiescholleck and Manahan-Vaughan, 2014). We have proposed that these changes in learning ability in middle-age relate less to a cognitive decline *per se*, but rather may reflect less flexibility in revising hard-wired knowledge or reduced adaptability to new learning challenges (Wiescholleck and Manahan-Vaughan, 2014). These age-related changes may be state-dependent: rats (as well as humans) can be expected to be less enthused in middle-age by stimuli that would have been perceived as very exciting in youth (“*been there, done that*”) and thus, higher excitation thresholds may need to be surpassed in driving the hippocampus towards the synaptic encoding of experience. The dopaminergic and noradrenergic systems are pivotal in the signaling of novelty, motivation and arousal and both noradrenaline and dopamine release in the hippocampus drives elevations of neuronal excitability (Harley, 2007; Hamilton et al., 2010). We speculated that the loss of learning ability in middle-age may relate to changes in neuromodulatory control of the hippocampus by these components of the catecholaminergic system. We report here that although LTP is equivalent in young and middle-aged animals, and although middle-aged animals respond to a change in spatial environment with LTP, we detected a failure of dopamine D1/D5 receptor and beta-adrenergic receptor agonists to facilitate weak potentiation into persistent (>24 h) LTP, as was the case in young rats.

## Materials and Methods

The present study was carried out in accordance with the European Communities Council Directive of 22 September 2010 (2010/63/EU) for care of laboratory animals and after approval of the local government ethics committee (Landesamt für Naturschutz, Umweltschutz und Verbraucherschutz, Nordrhein Westfalen). All efforts were made to minimize the number of animals used.

### Electrophysiology

Seven- to eight-week old male Wistar rats (Charles River, Germany) were anesthetized using sodium pentobarbital, (52 mg/kg, intraperitoneal, i.p., injection). Stimulating and recording electrodes were implanted as described previously (Hansen and Manahan-Vaughan, 2015b). A bipolar stimulation electrode was placed in the medial perforant path (6.9 mm posterior to bregma and 4.1 mm lateral to the midline). An unipolar recording electrode was implanted into the dentate gyrus (3.1 mm posterior to bregma and 1.9 mm lateral to the midline). The electrode depths were determined by means of electrophysiological recordings during the implantation procedure. A guide cannula was implanted to allow injections into the intracerebral ventricle (i.c.v.;Manahan-Vaughan, 1997). The coordinates used were: 0.5 mm posterior to bregma, 1.6 mm lateral to the midline (size: 5.6 mm length, 0.8 mm diameter, 4.5 mm depth).

The animals recovered for 7–10 days after surgery, before experiments were started. During experiments the animals could move freely in a recording chamber (40 × 40 × 50 cm) and had *ad libitum* access to water. Food intake was mildly restricted to maintain the animal’s body weight in a healthy range during aging. Two separate cohorts of animals were used (2–4 month and 8–14 month old rats), whereby both cohorts underwent electrode implantation at 7–8 weeks postnatally. Post-mortem histological assessment of electrode and cannula placements was conducted and animals with incorrect placements or anatomical disturbances were excluded from the study.

Experiments were conducted with at least 7 days hiatus between protocols. Evoked potentials were obtained and recorded as described previously (Hansen and Manahan-Vaughan, 2015a). Each time-point consisted of an average of five consecutive potentials triggered by test-pulse stimulation at 0.025 Hz.

LTP or STP was induced either by high-frequency stimulation (HFS) or submaximal weak high-frequency stimulation (wHFS) of afferent fibers. This comprised 10 bursts (for LTP) or three bursts (for STP) of 15 pulses at 200 Hz, with a 10 s interburst interval.

### Compounds

The D1/D5 receptor agonist, chloro-PB (Sigma-Aldrich, Taufkirchen, Germany) and the beta-adrenergic receptor agonist, isoproterenol (Tocris Bioscience, Bristol, UK), were dissolved in 0.9% NaCl. The agonists, or vehicle were applied in a 5 μl volume, as an intracerebral (*icv*) injection, to the lateral cerebral ventricle, that was delivered gradually over a 5 min period via a Hamilton syringe (Hamilton Company, Reno, NV, USA). All injections were carried out 30 min before HFS/wHFS was applied. Prior to this, 30 min recording of basal synaptic responses using test-pulse stimulation was conducted. Where effects of the compounds on basal synaptic transmission were assessed, the same experimental structure and timeline were followed, with the exception that HFS/wHFS was not applied.

### Holeboard Presentation

Presentation of a novel spatial environment in the form of an empty holeboard was conducted using previously described procedures and apparatus (Kemp and Manahan-Vaughan, 2004, 2008a). This particular protocol involves a spatial learning experience that reinforces hippocampal LTP only if the experience is novel (Kemp and Manahan-Vaughan, 2004, 2008a; Hagena and Manahan-Vaughan, 2011). For this, a novel holeboard (39.5 × 39.5 cm, gray polyvinyl chloride) was inserted onto the floor of the recording chamber 30 min after drug application, and was left there for 10 min. Directly after inserting the holeboard wHFS was applied. Animals that did not explore the holeboard or that showed freezing behavior were excluded from the experiment. Behavior was video-recorded to allow subsequent analysis.

### Data Analysis

The first 30 min of recording (6 time-points) were used as a reference “baseline”, and all time-points were expressed as the mean percentage ± the standard error of the mean (SEM) of this average baseline result. Recordings were obtained every 5 min until 15 min after HFS (or wHFS, or wHFS + holeboard) and then every 15 min until 4 h had elapsed. The following day an additional 60 min of recordings were made (24 h values). For analysis of differences between groups, multifactorial analysis of variance (ANOVA) with repeated measures was used. A *post hoc* Student’s *t*-test was used to assess the significance of individual time-points. The level of significance were set at *p* < 0.05.

## Results

### Basal Synaptic Transmission in The Dentate Gyrus of Middle-Aged Rats is not Influenced by Agonist Activation of D1/D5 or Beta-Adrenergic Receptors

**Figure 1:**
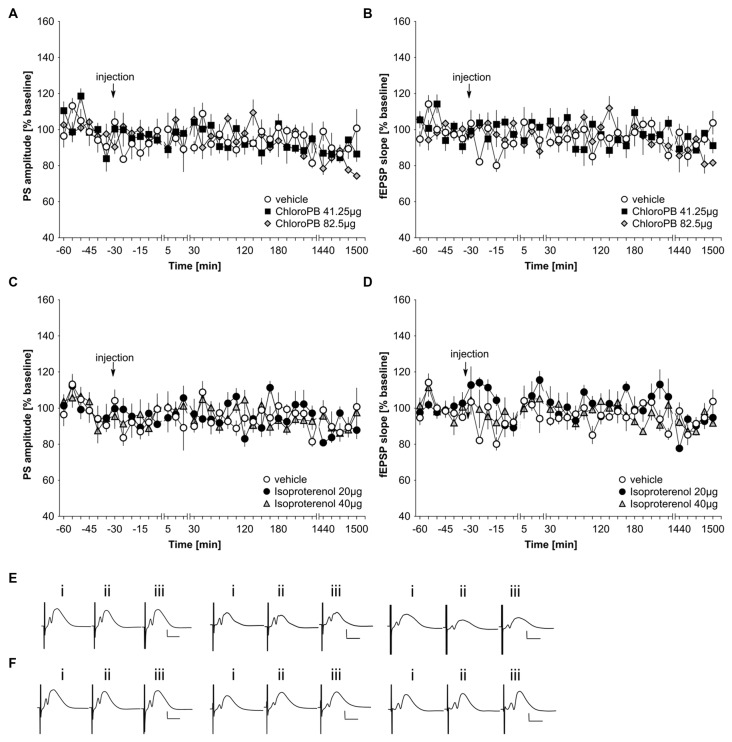
**Basal synaptic transmission in the dentate gyrus of middle-aged rats is not influenced by agonist activation of D1/D5 or beta-adrenergic receptors.** In vehicle-treated middle-aged animals, test-pulse stimulation evoked stable fEPSP and population spike (PS) responses for the duration of the monitoring period. **(A,B)** Treatment with the D1/D5 receptor agonist, chloro-PB (41.25 μg, 82.5 μg) has no effect on the PS **(A)** or the fEPSP **(B)** compared to vehicle-treated controls. **(C,D)** Treatment with the beta-adrenergic receptor agonist, isoproterenol (20 μg, 40 μg) has no effect on the PS **(C)** or the fEPSP **(D)** compared to vehicle-treated controls. The arrow in the graphs indicates time-point of injection. Line-breaks indicate changes in time-scale. **(E)** Analog examples depict potentials obtained in a vehicle experiment (left traces), in the presence of 41.25 μg of chloroPB (Middle traces) or 82.5 μg chloroPB (right traces) at: (i) 5 min prior to injection; (ii) 5 min post-injection; and (iii) 24 h post-injection. Vertical scale bar: 2 mV, horizontal scale bar: 8 ms. **(F)** Analog examples depict potentials obtained in a vehicle experiment (left traces), in the presence of 20 μg of isoproterenol (Middle traces) or 40 μg isoproterenol (right traces) at: (i) 5 min prior to injection; (ii) 5 min post-injection; and (iii) 24 h post-injection. Vertical scale bar: 2 mV, horizontal scale bar: 8 ms.

In previous studies, we reported that *icv* application of the dopamine D1/D5 agonist, chloro-PB (41.25 μg), has no effect on basal synaptic transmission in the dentate gyrus of freely behaving young rats (Wiescholleck and Manahan-Vaughan, 2014). Here, we tested the same dose on middle-aged (8–14 month old) rats (*n* = 5). The higher chloro-PB dose of 82.5 μg (*n* = 5) was also tested. We observed no significant group effect on population spike (PS) or fEPSP responses as a result of treatment with either agonist dose compared to vehicle-injected middle-aged rats (Figures 1A,B,E; ANOVA group effect, PS: *F*_(2,12)_ = 0.22, *p* = 0.80; fEPSP: *F*_(2,12)_ = 0.15; *p* = 0.86).

Previously, we also showed that the beta-adrenergic receptor agonist, isoproterenol at a dose of 20 μg does not affect basal synaptic transmission in CA1 of freely behaving young rats (Kemp and Manahan-Vaughan, 2008b). In the dentate gyrus neither a dose of 20 μg (*n* = 5), nor a dose of 40 μg (*n* = 5), affected basal synaptic transmission compared to vehicle-treated middle-aged rats (*n* = 5; Figures 1C,D,F; ANOVA group effect, PS: *F*_(2,12)_ = 0.2, *p* = 0.98; fEPSP: : *F*_(2,12)_ = 1.39, *p* = 0.29).

### Agonist Activation of Dopamine D1/D5 Receptors Prolongs Synaptic Potentiation in The Dentate Gyrus of Young But not Middle-Aged Rats

**Figure 2:**
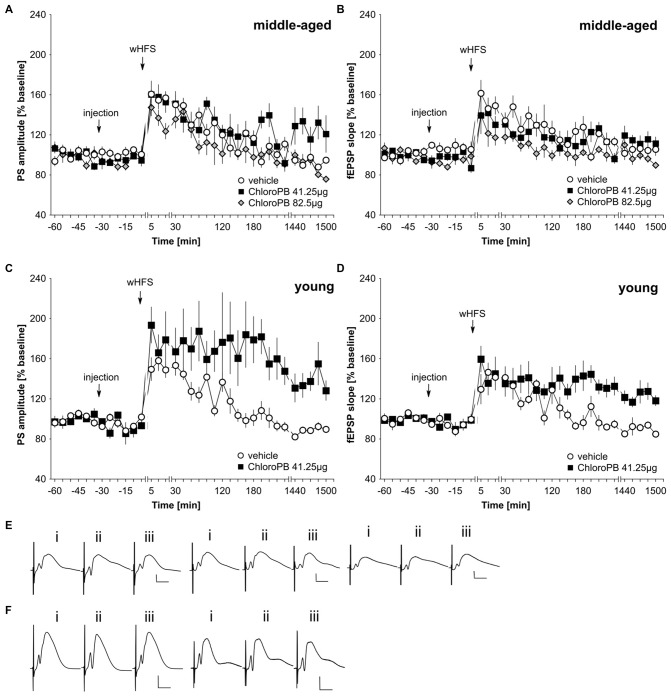
**Agonist activation of dopamine D1/D5 receptors prolongs synaptic potentiation in the dentate gyrus of young but not middle-aged rats. (A,B)** In vehicle-treated middle-aged animals weak high-frequency stimulation (wHFS) results in short-term potentiation (STP) of PS **(A)** and fEPSP **(B)** that lasted for *ca.* 2 h. When wHFS was applied in the presence of the D1/D5-receptor agonist chloroPB (41.25 μg, 82.5 μg) STP occurs that is not significantly different from vehicle-treated controls. **(C,D)** Vehicle-treated young animals that receive wHFS, respond with STP of PS **(C)** and fEPSP **(D)** that lasts for *ca.* 2 h, whereas wHFS given in the presence of chloroPB (41.25 μg) results in long-term potentiation (LTP) that lasts for at least 25 h. The arrows in the graphs indicate the time-point of injection, or wHFS. Line-breaks indicate changes in time-scale. **(E)** Analog examples depict potentials that were evoked during a control experiment (left traces), in the presence of 41.25 μg of chloroPB (Middle traces) or 82.5 μg chloroPB (right traces) in middle-aged animals. Potentials were obtained: (i) 5 min prior to wHFS; (ii) 5 min post-wHFS; and (iii) 24 h post-wHFS. Vertical scale bar: 2 mV, horizontal scale bar: 8 ms. **(F)** Analog examples depict potentials obtained during a vehicle experiment (left traces), or in the presence of 41.25 μg of chloroPB (right traces) in young animals, Responses were obtained: (i) 5 min prior to wHFS; (ii) 5 min post-wHFS; and (iii) 24 h post-wHFS. Vertical scale bar: 2 mV, horizontal scale bar: 8 ms.

wHFS, three bursts of 15 pulses at 200 Hz induced STP in control animals (*n* = 6; Figures 2A,B,E). When we applied the same wHFS protocol in the presence of chloro-PB (41.25 μg), we observed that STP resulted that was not significantly different from vehicle-treated controls (*n* = 6; Figures 2A,B,E; ANOVA group effect, PS: *F*_(1,10)_ = 2.25, *p* = 0.16; fEPSP: : *F*_(1,10)_ = 0.40, *p* = 0.54). Raising the dose to 82.5 μg (*n* = 5) failed to alter the outcome of the result: STP was elicited that was still equivalent to controls (*n* = 6; Figures 2A,B,E; ANOVA group effect, PS: *F*_(1,9)_ = 2.95, *p* = 0.12; interaction effect: *F*_(22,198)_ = 0.23, *p* = 0.23). A tendency towards reduced fEPSP values was evident (group effect: *F*_(1,9)_ = 7.65, *p* = 0.02), but this was absent with regard to an interaction effect (ANOVA: *F*_(22,198)_ = 0.85, *p* = 0.67).

Prior studies showed that HFS when given in the presence of chloro-PB results in a prolongation of STP into LTP (Li et al., 2003; Lemon and Manahan-Vaughan, 2006). We were concerned that the lack of effect of the agonist in our middle-aged animals could relate to subtle differences in the responsiveness of the strain of rat used (Manahan-Vaughan, 2000). Thus, to verify that this property was evident in our young animals, we examined the effect of applying the STP-inducing stimulation protocol in the presence of chloro-PB (41.25 μg) in 2–4 month old rats (*n* = 6). Here, we observed a significant facilitation of LTP compared to vehicle-treated young rats (*n* = 6; Figures 2C,D,F; ANOVA PS: *F*_(1,10)_ = 7.56, *p* = 0.02; fEPSP: group effect: *F*_(1,10)_ = 9.97, *p* = 0.01).

### Agonist Activation of Beta-Adrenergic Receptors Prolongs Synaptic Potentiation in The Dentate Gyrus of Young But not Middle-Aged Rats

**Figure 3:**
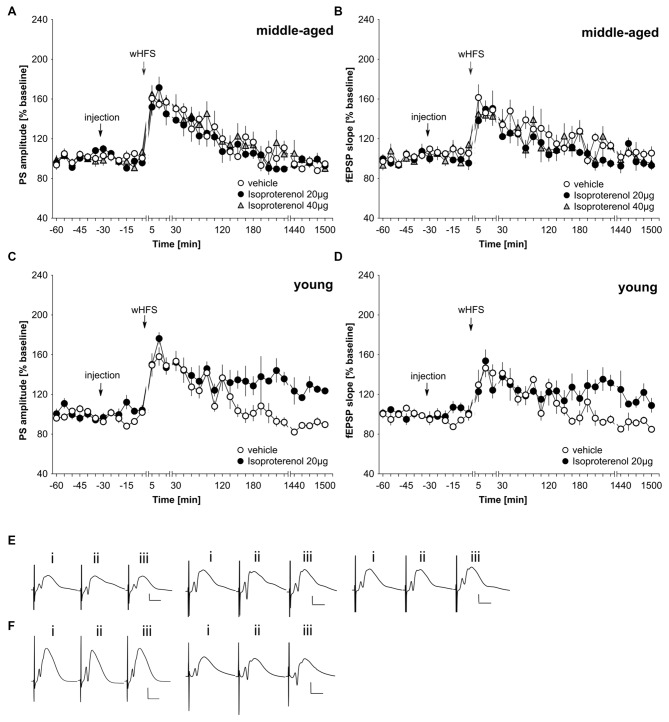
**Agonist activation of beta-adrenergic receptors prolongs synaptic potentiation in the dentate gyrus of young, but not middle-aged, rats. (A,B)** In vehicle-treated middle-aged animals wHFS results in STP of PS **(A)** and fEPSP **(B)** that lasts for *ca.* 2 h. wHFS in the presence of the beta-adrenergic receptor agonist, isoproterenol (20 μg, 40 μg), results in STP that is not significantly different from vehicle-treated controls. Line breaks indicate change in time scale. **(C,D)** Vehicle-treated young animals that received wHFS, express STP of PS **(C)** and fEPSP **(D)** that lasts for *ca.* 2 h. wHFS in the presence of isoproterenol (20 μg) results in LTP that lasts for at least 25 h. The arrows in the graphs indicate the time-point of injection, or wHFS. Line-breaks indicate changes in time-scale. **(E)** Analog examples depict potentials from vehicle experiment (left traces), in the presence of 20 μg of isoproterenol (Middle traces) or 40 μg isoproterenol (right traces) in middle-aged animals obtained: (i) 5 min prior to wHFS; (ii) 5 min post-wHFS; and (iii) 24 h post-wHFS. Vertical scale bar: 2 mV, horizontal scale bar: 8 ms. **(F)** Analog examples depict potentials from vehicle experiment (left traces), in the presence of 20 μg of isoproterenol (right traces) in young animals obtained: (i) 5 min prior to wHFS; (ii) 5 min post-wHFS; and (iii) 24 h post-wHFS. Vertical scale bar: 2 mV, horizontal scale bar: 8 ms.

In freely behaving rats, agonist activation of beta-adrenergic receptors by means of *icv* application of 20 μg isoproterenol results in a prolongation of STP into LTP that lasts for at least 24 h (Hansen and Manahan-Vaughan, 2015b). We first assessed whether this dose of agonist could facilitate LTP in middle-aged rats. Here, we observed that 20 μg of isoproterenol (*n* = 6) had no significant impact on STP compared to STP elicited in vehicle-treated controls (*n* = 6; Figures 3A,B,E; ANOVA PS, group effect: *F*_(1,10)_ = 0.81, *p* = 0.39; PS, interaction effect: *F*_(22,220)_ = 1.18, *p* = 0.27; fEPSP group effect: *F*_(1,10)_ = 3.35, *p* = 0.1; fEPSP interaction effect: *F*_(22,220)_ = 0.91, *p* = 0.59). Raising the dose to 40 μg of isoproterenol (*n* = 6) failed to alter the profile of STP elicited (Figures 3A,B,E). (ANOVA, PS group effect: *F*_(1,10)_ = 0.44, *p* = 0.52; PS interaction effect: *F*_(22,220)_ = 0.76, *p* = 0.78; fEPSP group effect: *F*_(1,10)_ = 0.92, *p* = 0.36; fEPSP interaction effect: *F*_(22,220)_ = 0.94, *p* = 0.55).

Given the lack of effect of the agonist on STP in our middle-aged rats, we checked that isoproterenol facilitated STP into LTP in young rats, as had been previously reported (Hansen and Manahan-Vaughan, 2015b). Here, in 2–4 month old rats, we could confirm that 20 μg of isoproterenol (*n* = 6) significantly prolongs STP into LTP, compared to STP that is evoked in vehicle-treated young rats (*n* = 6; Figures 3C,D,F; ANOVA, PS group effect: *F*_(1,10)_ = 26.08, *p* = 0.0005; PS interaction effect: *F*_(22,220)_ = 1.89, *p* = 0.011; fEPSP group effect: *F*_(1,10)_ = 3.83, *p* = 0.08; fEPSP interaction effect: *F*_(22,220)_ = 2.55, *p* = 0.0003).

### LTP is Equivalent in Middle-Aged And Young Rats. Novel spatial learning facilitates LTP expression in middle-aged rats

**Figure 4:**
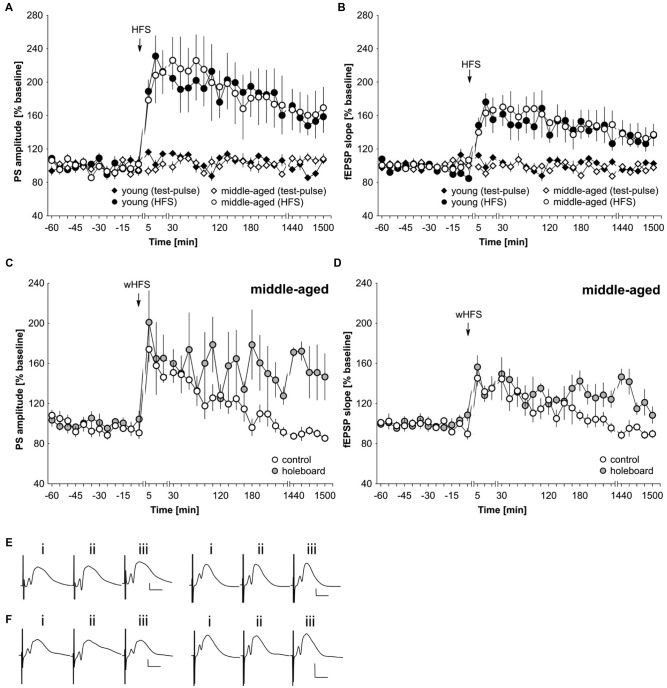
**LTP is unaltered in middle-aged compared to young rats.** Novel spatial learning facilitates LTP expression in middle-aged rats. **(A,B)** Strong high-frequency stimulation (HFS) results in LTP of the PS **(A)** and fEPSP **(B)** that lasts for at least 25 h in both young and middle-aged rats. Test-pulse stimulation results in equivalently stable basal synaptic transmission in both age groups. **(C,D)** wHFS resulted in STP of the PS **(C)** and fEPSP **(D)** in the dentate gyrus of middle aged rats. Coupling wHFS with novel spatial exploration of a holeboard facilitated STP into LTP that lasted for at least 25 h. The arrow in the graphs indicates the time-point of HFS/wHFS. Line-breaks indicate changes in time-scale. **(E)** Analog examples depict potentials from young animals (left traces) and middle-aged animals (right traces): (i) 5 min prior to HFS; (ii) 5 min post-HFS; and (iii) 24 h post-HFS. Vertical scale bar: 2 mV, horizontal scale bar: 8 ms. **(F)** Analog examples depict potentials from middle-aged animals that received wHFS only (left traces) and from middle-aged animals that received wHFS during novel holeboard exploration (right traces) obtained: (i) 5 min prior to wHFS; (ii) 5 min post-wHFS; and (iii) 24 h post-wHFS. Vertical scale bar: 2 mV, horizontal scale bar: 8 ms.

The lack of facilitation by agonist activation of D1/D5 and beta-adrenergic receptors of STP could result from generalized deficits in synaptic plasticity in middle-aged rats. To assess this possibility, we applied a strong tetanization protocol that typically results in persistent (>24 h) LTP in the dentate gyrus of young freely behaving rats (Twarkowski et al., 2016). HFS (200 Hz, 10 × 15 pulses) resulted in robust LTP in both young (*n* = 6) and middle-aged (*n* = 6) rats that did not differ significantly in profile or magnitude (Figures 4A,B,E; ANOVA, PS group effect: *F*_(1,10)_ = 0.01, *p* = 0.94; PS interaction effect: *F*_(22,220)_ = 0.64, *p* = 0.89; fEPSP group effect: *F*_(1,10)_ = 0.06, *p* = 0.82; fEPSP interaction effect: *F*_(22,220)_ = 0.62, *p* = 0.91). Basal synaptic transmission was also equivalent in young (*n* = 6) and middle-aged rats (*n* = 6; Figures 4A,B,E).

Novel exploration of new space facilitates STP into LTP in the dentate gyrus of young rats (Kemp and Manahan-Vaughan, 2008b). Here, we also assessed whether this property is evident in middle-aged rats. The coupling of novel spatial exploration with weak HFS (wHFS, 200 Hz, 3 × 15 pulses) resulted in robust LTP that lasted for over 25 h in middle-aged rats (*n* = 5) compared to animals that received wHFS only (*n* = 5; Figures 4C,D,F; ANOVA group effect, PS: *F*_(1,8)_ = 4.05; *p* = 0.08; interaction effect, PS: *F*_(22,176)_ = 1.87, *p* = 0.0138; group effect fEPSP: *F*_(1,8)_ = 3.98, *p* = 0.08; interaction effect fEPSP: *F*_(22,176)_ = 1.80, *p* = 0.0198). The most potent effects occurred at the late phase of LTP (*t*-test 24–25 h post-wHFS, PS: *t*_(4)_ = −3.77, *p* = 0.0196; fEPSP: *t*_(4)_ = −4.37, *p* = 0.0119).

## Discussion

In this study, we show that agonist activation of dopamine D1/D5 receptors, or of beta-adrenergic receptors, fail to prolong STP into LTP in the dentate gyrus of middle-aged rats, even though these doses are effective in young animals. This absence of effect does not appear to relate to putative deficits in hippocampal plasticity: a stronger tetanization protocol is successful in inducing persistent (>24 h) LTP that is equivalent in profile and magnitude to that elicited in young animals. Furthermore, the coupling of weak afferent stimulation with novel spatial exploration prolongs STP into LTP in middle-aged animals, as has been previously reported for young animals (Kemp and Manahan-Vaughan, 2008a). Taken together, these results indicate that, with increasing age, the dopaminergic and noradrenergic systems become less effective in supporting memory encoding through LTP. This may relate to state-dependent changes in the activation of the catecholaminergic system.

Receptors, such as the NMDA, dopamine D1/D5 and beta-adrenergic receptors, are important elements in mediating synaptic plasticity (Herron et al., 1986; Frey et al., 1990; Pöschel and Manahan-Vaughan, 2007; Hansen and Manahan-Vaughan, 2015b; Chay et al., 2016). Male rats show lower hippocampal NMDA receptor subunit expression and decreased NMDA receptor function as early as 8–14 months postnatally (Monfort and Felipo, 2007; Guidi et al., 2015). Although, NMDA receptors are important in initiating synaptic potentiation (Herron et al., 1986; Pöschel and Manahan-Vaughan, 2007) we did not find any difference in the magnitude or duration in the early or late phase of LTP induced by high frequency afferent stimulation (HFS) in middle-aged animals, compared to young adult animals. This finding is in line with other studies of LTP in the dentate gyrus of middle-aged rats (Barnes et al., 2000). This suggests that at this stage of maturity, changes in NMDA receptor levels are not yet adequate to mediate a change in LTP. Alternatively compensatory mechanisms are already underway: in the dentate gyrus, LTP induction is also supported by L-type voltage gated Ca^2+^ channels (Manahan-Vaughan et al., 1998).

Both dopamine and noradrenaline release in the hippocampus results in an increase of neuronal excitability in the dentate gyrus that predisposes this hippocampal subfield towards expression of LTP (Harley, 2007; Hamilton et al., 2010). Dopamine D1/D5 and beta-adrenergic receptors are also highly important for the maintenance of the later phases of LTP in young adult rodents (Stanton and Sarvey, 1985; Straube and Frey, 2003; Hansen and Manahan-Vaughan, 2015b). In the present study we found that pharmacological activation of either dopamine D1/D5 or beta-adrenergic receptors fail to facilitate LTP in middle-aged animals. Both dopamine D1/D5 and beta-adrenergic receptors exhibit an age-dependent decrease in ligand binding and reduced amount of total receptor expression in the hippocampus that is evident at 12 months (Popova and Petkov, 1989; Araki et al., 1997; Suzuki et al., 2001). An inhomogeneous shift in D1 receptor expression has been reported in 12–24 month old rats compared to young (3 month old) adults, however (Amenta et al., 2001): here, increases in receptor expression emerged in the CA1 region and decreases occurred in the dentate gyrus. This could explain why agonist activation of D1/D5 receptors was ineffective in modulating dentate gyrus LTP in middle-aged rats, as seen in our study. Interestingly, another study (Reis et al., 2005) showed that not only beta-adrenergic receptor mediated signaling, but also adenylate-cyclase mediated signaling is directly impaired in 24 month old animals. Therefore, the lack of LTP facilitation by dopamine D1/D5 receptor, or beta-adrenergic receptor agonists in middle-aged animals, that we observed, might reflect the gradual development of changes in receptor expression/agonist binding as well as in signaling efficacy through adenylate cyclase. Since receptors of the dopaminergic and noradrenergic systems have been proposed to act synergistically with NMDA receptors to enable long-lasting synaptic plasticity (Kauer et al., 1988; Frey et al., 1990; Chay et al., 2016), changes in NMDA receptor signaling may also contribute to these effects.

In young rats, both patterned stimulation of hippocampal afferents and novel spatial exploration induce an increase in extracellular levels of noradrenaline and dopamine in the hippocampus (Ihalainen et al., 1999; Neugebauer et al., 2009), most likely through increased activity of the locus coeruleus and ventral tegmental area (Sara et al., 1994; Horvitz et al., 1997; Kitchigina et al., 1997; Lemon et al., 2009). In healthy rodents, locus coeruleus projections to diverse brain structures begin to decline by 7–15 months of age (Ishida et al., 2000; Shirokawa et al., 2000). Compensatory mechanisms in the form of increased axonal branching and physiological changes of those projections have been described (Ishida et al., 2000; Shirokawa et al., 2000). Similarly, age-related changes in the ventral tegmental area have been reported (Schuligoi et al., 1993; Siddiqi et al., 1999). The locus coeruleus and ventral tegmental area are anatomically and functionally connected (Simon et al., 1979; Ornstein et al., 1987). Lesions of one of those nuclei increases the vulnerability of the other nucleus (Szot et al., 2012), which suggests that aging-related deficits in the locus coeruleus may have an impact on ventral tegmental area function. In 22–24 month old rats decreased synthesis of noradrenaline and, to a lesser extent, dopamine have been described (Luine et al., 1990; Dickerson et al., 2009). Taking into account the necessity for dopamine D1/D5 and beta-adrenergic receptor activation for hippocampus-dependent learning (Straube et al., 2003; Hansen and Manahan-Vaughan, 2015a, b), age-dependent reductions in noradrenaline and dopamine levels are likely to lead to deficits in spatial learning. In line with this, middle-aged rats exhibit deficits in hippocampus-related learning such as context-dependent renewal of extinguished memory, or object-place memory (Cavoy and Delacour, 1993; Wiescholleck et al., 2014). A decrease in attention and vigilance is also evident in middle-aged rats (Jones et al., 1995; Guidi et al., 2015). Our observation that a loss of sensitivity of dopamine D1/D5 and beta-adrenergic receptors occurs in LTP processes in middle-age may comprise a cellular mechanism that underlies these behavioral changes.

Interestingly, LTP appears unchanged in its magnitude and profile in middle-aged animals compared to young adults. We found this to be the case both for LTP that is induced by strong tetanic stimulation of hippocampal afferents, and for LTP that is facilitated by the conjunction of weak afferent stimulation and novel spatial learning. In line with this, other *in vivo* studies of dentate gyrus LTP reported that 6–12 month old F344 rats express robust LTP that lasts for several days, although 22–30 month old rats show impaired, or no, LTP expression (Orr et al., 2001; Sierra-Mercado et al., 2008). Both forms of persistent LTP that we examined are likely to be related to experience-dependent memory encoding (Martin et al., 2000; Straube and Frey, 2003; Kemp and Manahan-Vaughan, 2008a). Age-dependent changes in LTP may not occur in synchrony across the different hippocampal subfields, however: a decrease in LTP magnitude at Schaffer-Collateral CA1 synapses of 6 month vs. 22 month old rats (Monfort and Felipo, 2007) and a loss of late (>1 day) LTP at perforant path-CA3 synapses in 2 month vs. 8 month old rats (Dieguez and Barea-Rodriguez, 2004) have been reported. These subfield-specific alterations may perhaps explain why middle-aged rats show deficits in specific forms of hippocampus-dependent memory (Wiescholleck et al., 2014).

It is also striking that spatial novelty remained effective in promoting weak potentiation into persistent LTP in middle-aged rats, despite the fact that the D1/D5 and beta-adrenergic receptor agonists failed to pharmacologically enhance STP into LTP in this age-group. Taken together with our observation that strong afferent stimulation resulted in persistent LTP in the middle-aged animals, this finding suggests that if a behavioral (or synaptic) impetus is salient enough, LTP will result in these older rats. Transduction of behavioral saliency into cellular signals that promote hippocampal information encoding is very likely to be mediated by the noradrenergic and dopaminergic systems (Hansen and Manahan-Vaughan, 2014; Hagena et al., 2016). So why then, does agonist activation of these receptors fail to strengthen weak potentiation in the middle-aged animals? Learning-facilitated LTP is strongly dependent on the activation of both of these receptors in younger rats (Kemp and Manahan-Vaughan, 2008a; Hagena and Manahan-Vaughan, 2012; Lemon and Manahan-Vaughan, 2012). As mentioned above, one possible explanation for the lack of effect of D1/D5 and beta-adrenergic receptor activation on STP in the older rats may relate to changed expression of the catecholamine receptors that develops with aging (Popova and Petkov, 1989; Araki et al., 1997; Suzuki et al., 2001). Prolonged agonist activation of the receptors may serve to desensitize, or promote tachyphylaxis of, a significant pool of those receptors that still remain functional in aging animals (Benovic et al., 1988; Bouvier et al., 1988; Hausdorff et al., 1989; Ng et al., 1994), thereby negating the kind of positive effects on LTP that are detectable (following agonist treatment) in younger animals. However, it may also be the case that the *temporal* pattern of catecholamine receptor activation that is enabled by means of burst firing of the locus coeruleus (Sara, 2015) and/or ventral tegmental area (Schultz, 1998; Bromberg-Martin et al., 2010; Paladini and Roeper, 2014) is decisive in enabling synaptic information encoding. Whereas the lack of effect of the agonists in middle-aged animals may herald the onset of deficits in catecholamine receptor-related hippocampal information processing, the retention of the ability to respond to spatial novelty with persistent LTP suggests that, at this time-point in aging, the catecholaminergic system has not yet become entirely dysfunctional.

Changes in object-recognition memory and in context-dependent extinction learning become apparent in middle-aged rodents (Wiescholleck et al., 2014). With increasing age, exploratory behavior of rats also declines, and although object-place memory is impaired, older rats detect novelty related to newly included environmental features (Shukitt-Hale et al., 2001). Older rats are more vulnerable to distraction than younger animals (Mishra et al., 2014). This kind of attention deficit can be ameliorated by training, however (Mishra et al., 2014). These findings are in line with our interpretation that early age-related deficits in hippocampal information processing may less relate to a failure of learning *per se* and more to a decline in motivation and attention. This suggests that neuromodulatory systems that support attention, such as the dopaminergic and noradrenergic systems are not completely disabled, but may be become increasingly ineffective with aging. Their involvement in raising hippocampal excitability during e.g., novel experience and in lowering the thresholds for induction of hippocampal synaptic plasticity (Sara et al., 1994; Li et al., 2003; Lemon et al., 2009; Hansen and Manahan-Vaughan, 2015a, b) becomes progressively weaker, however, necessitating that a higher degree of arousal (than that required in young animals) must be elicited in order for the animal to learn effectively.

## Conclusion

In young rats, the dopaminergic and noradrenergic systems exert a very potent control on the longevity and magnitude of LTP (Hansen and Manahan-Vaughan, 2014; Hagena et al., 2016). This regulation is very tightly linked to hippocampus-based learning: antagonism of dopamine D1/D5 receptors prevents the facilitation of long-term depression (LTD) by spatial content learning in the CA1 region (Lemon and Manahan-Vaughan, 2006) and the dentate gyrus (Wiescholleck and Manahan-Vaughan, 2014). The dopamine D1/D5 receptors are required for very long-lasting forms of LTP and LTD in behaving rats (Kulla and Manahan-Vaughan, 2000; Lemon and Manahan-Vaughan, 2006), as are beta-adrenergic receptors (Kemp and Manahan-Vaughan, 2008b; Hagena and Manahan-Vaughan, 2012; Hansen and Manahan-Vaughan, 2015a, b). Thus, it was all the more surprising to find that activation of neither receptor could prolong LTP in middle-aged rats. This deficit does not appear to relate to failure of LTP machinery: strong afferent stimulation resulted in LTP that was equivalent in young and middle-aged rats. Furthermore, weak afferent stimulation during novel spatial exploration resulted in LTP. This latter phenomenon has been extensively described in young rats (Kemp and Manahan-Vaughan, 2008a). The facilitation of LTP by novel spatial exploration is very tightly linked to the spatial learning event (Kemp and Manahan-Vaughan, 2008a) and is prevented in young animals by antagonism of either D1/D5 receptors (Wiescholleck and Manahan-Vaughan, 2014) or beta-adrenergic receptors (Kemp and Manahan-Vaughan, 2008b; Hagena and Manahan-Vaughan, 2012; Hansen and Manahan-Vaughan, 2015b) that prevent the learning event itself. The fact that robust LTP can be either induced by afferent stimulation or by spatial learning in middle-aged rats suggests that the ability of the hippocampus to express persistent LTP is intact. Changes in the sensitivity of the catecholaminergic receptors to their respective agonists (Suzuki et al., 2001) may reflect a more subtle loss of the state-dependent regulation of learning and synaptic encoding by these neuromodulatory systems. This desensitization of hippocampal LTP to catecholaminergic control may reflect the functional basis for changes in attention and motivation-based learning that emerge with increasing age.

## Author Contributions

DM-V created the concept and strategy of the study. HT conducted the electrophysiological experiments and prepared the corresponding figures. Data analysis and interpretation were conducted jointly. DM-V wrote the article, with contributions from HT.

## Conflict of Interest Statement

The authors declare that the research was conducted in the absence of any commercial or financial relationships that could be construed as a potential conflict of interest.

## References

[B1] Almaguer-Melian W. Rojas-Reyes Y. Alvare A. Rosillo J. C. Frey J. U. Bergado J. A. (2005). Long-term potentiation in the dentate gyrus in freely moving rats is reinforced by intraventricular application of norepinephrine, but not oxotremorine. Neurobiol. Learn. Mem. 83, 72–78. 10.1016/j.nlm.2004.08.002 15607691

[B2] Amenta F. Mignini F. Ricci A. Sabbatini M. Tomassoni D. Tayebati S. K. (2001). Age-related changes of dopamine receptors in the rat hippocampus: a light microscope autoradiography study. Mech. Ageing Dev. 122, 2071–2083. 10.1016/s0047-6374(01)00317-7 11589924

[B3] Araki T. Kato H. Shuto K. Fujiwara T. Itoyama Y. (1997). Effect of aging on dopaminergic receptors and uptake sites in the rat brain studied by receptor autoradiography. J. Neurol. Sci. 148, 131–137. 10.1016/s0022-510x(96)05343-9 9129108

[B4] Aston-Jones G. Bloom F. E. (1981). Norepinephrine-containing locus coeruleus neurons in behaving rats exhibit pronounced responses to non-noxious environmental stimuli. J. Neurosci. 1, 887–900. 7346593 10.1523/JNEUROSCI.01-08-00887.1981 PMC6564231

[B5] Ballesteros S. Bischof G. N. Goh J. O. Park D. C. (2013). Neural correlates of conceptual object priming in young and older adults: an event-related functional magnetic resonance imaging study. Neurobiol. Aging 34, 1254–1264. 10.1016/j.neurobiolaging.2012.09.019 23102512 PMC4028122

[B6] Barnes C. A. Rao G. Houston F. P. (2000). LTP induction threshold change in old rats at the perforant path-granule cell synapse. Neurobiol. Aging 21, 613–620. 10.1016/s0197-4580(00)00163-9 11016529

[B7] Benovic J. L. Bouvier M. Caron M. G. Lefkowitz R. J. (1988). Regulation of adenylyl cyclase-coupled beta-adrenergic receptors. Annu. Rev. Cell Biol. 4, 405–428. 10.1146/annurev.cb.04.110188.002201 2848553

[B8] Bouvier M. Hausdorff W. P. De Blasi A. O’Dowd B. F. Kobilka B. K. Caron M. G.. (1988). Removal of phosphorylation sites from the β_2_-adrenergic receptor delays onset of agonist-promoted desensitization. Nature 333, 370–373. 10.1038/333370a0 2836733

[B9] Bromberg-Martin E. S. Matsumoto M. Hikosaka O. (2010). Dopamine in motivational control: rewarding, aversive and alerting. Neuron 68, 815–834. 10.1016/j.neuron.2010.11.022 21144997 PMC3032992

[B10] Cavoy A. Delacour J. (1993). Spatial but not object recognition is impaired by aging in rats. Physiol. Behav. 53, 527–530. 10.1016/0031-9384(93)90148-9 8451318

[B11] Chay A. Zamparo I. Koschinski A. Zaccolo M. Blackwell K. T. (2016). Control of βAR- and *N*-methyl-*D*-aspartate (NMDA) receptor-dependent cAMP dynamics in hippocampal neurons. PLoS Comput. Biol. 12:e1004735. 10.1371/journal.pcbi.1004735 26901880 PMC4763502

[B12] Dickerson J. W. Hemmerle A. M. Numan S. Lundgren K. H. Seroogy K. B. (2009). Decreased expression of ErbB4 and tyrosine hydroxylase mRNA and protein in the ventral midbrain of aged rats. Neuroscience 163, 482–489. 10.1016/j.neuroscience.2009.06.008 19505538 PMC2755587

[B13] Dieguez D. Barea-Rodriguez E. J. (2004). Aging impairs the late phase of long-term potentiation at the medial perforant path-CA3 synapse in awake rats. Synapse 52, 53–61. 10.1002/syn.20004 14755632 PMC1913478

[B14] Driscoll I. Hamilton D. A. Petropoulos H. Yeo R. A. Brooks W. M. Baumgartner R. N.. (2003). The aging hippocampus: cognitive, biochemical and structural findings. Cereb. Cortex 13, 1344–1351. 10.1093/cercor/bhg081 14615299

[B15] Frey U. Schroeder H. Matthies H. (1990). Dopaminergic antagonists prevent long-term maintenance of posttetanic LTP in the CA1 region of rat hippocampal slices. Brain Res. 522, 69–75. 10.1016/0006-8993(90)91578-5 1977494

[B16] Guidi M. Kumar A. Foster T. C. (2015). Impaired attention and synaptic senescence of the prefrontal cortex involves redox regulation of NMDA receptors. J. Neurosci. 35, 3966–3977. 10.1523/jneurosci.3523-14.2015 25740525 PMC4348191

[B17] Hagena H. Manahan-Vaughan D. (2011). Learning-facilitated synaptic plasticity at CA3 mossy fiber and commissural-associational synapses reveals different roles in information processing. Cereb. Cortex 21, 2442–2449. 10.1093/cercor/bhq271 21493717 PMC3183418

[B18] Hagena H. Manahan-Vaughan D. (2012). Learning-facilitated long-term depression and long-term potentiation at mossy fiber—CA3 synapses requires activation of β-adrenergic receptors. Front. Integr. Neurosci. 6:23. 10.3389/fnint.2012.00023 22654741 PMC3358719

[B19] Hagena H. Hansen N. Manahan-Vaughan D. (2016). β-adrenergic control of hippocampal function: subserving the choreography of synaptic information storage and memory. Cereb. Cortex 26, 1349–1364. 10.1093/cercor/bhv330 26804338 PMC4785955

[B20] Hamilton T. J. Wheatley B. M. Sinclair D. B. Bachmann M. Larkum M. E. Colmers W. F. (2010). Dopamine modulates synaptic plasticity in dendrites of rat and human dentate granule cells. Proc. Natl. Acad. Sci. U S A 107, 18185–18190. 10.1073/pnas.1011558107 20921404 PMC2964233

[B21] Hansen N. Manahan-Vaughan D. (2014). Dopamine D1/D5 receptors mediate informational saliency that promotes persistent hippocampal long-term plasticity. Cereb. Cortex 24, 845–858. 10.1093/cercor/bhs362 23183712 PMC3948488

[B22] Hansen N. Manahan-Vaughan D. (2015a). Locus coeruleus stimulation facilitates long-term depression in the dentate gyrus that requires activation of β-Adrenergic receptors. Cereb. Cortex 25, 1889–1896. 10.1093/cercor/bht429 24464942 PMC4459289

[B23] Hansen N. Manahan-Vaughan D. (2015b). Hippocampal long-term potentiation that is elicited by perforant path stimulation or that occurs in conjunction with spatial learning is tightly controlled by beta-adrenoreceptors and the locus coeruleus. Hippocampus 25, 1285–1298. 10.1002/hipo.22436 25727388 PMC6680149

[B24] Harley C. W. (2007). Norepinephrine and the dentate gyrus. Prog. Brain Res. 163, 299–318. 10.1016/s0079-6123(07)63018-0 17765726

[B25] Hausdorff W. P. Bouvier M. O’Dowd B. F. Irons G. P. Caron M. G. Lefkowitz R. J. (1989). Phosphorylation sites on two domains of the β_2_-adrenergic receptor are involved in distinct pathways of receptor desensitization. J. Biol. Chem. 264, 12657–12665. 2545714

[B26] Herron C. E. Lester R. A. Coan E. J. Collingridge G. L. (1986). Frequency-dependent involvement of NMDA receptors in the hippocampus: a novel synaptic mechanism. Nature 322, 265–268. 10.1038/322265a0 2874493

[B27] Horvitz J. C. Stewart T. Jacobs B. L. (1997). Burst activity of ventral tegmental dopamine neurons is elicited by sensory stimuli in the awake cat. Brain Res. 759, 251–258. 10.1016/s0006-8993(97)00265-5 9221945

[B28] Ihalainen J. A. Riekkinen P. Jr. Feenstra M. G. P. (1999). Comparison of dopamine and noradrenaline release in mouse prefrontal cortex, striatum and hippocampus using microdialysis. Neurosci. Lett. 277, 71–74. 10.1016/S0304-3940(99)00840-X 10624812

[B29] Ishida Y. Shirokawa T. Miyaishi O. Komatsu Y. Isobe K. (2000). Age-dependent changes in projections from locus coeruleus to hippocampus dentate gyrus and frontal cortex. Eur. J. Neurosci. 12, 1263–1270. 10.1046/j.1460-9568.2000.00017.x 10762355

[B30] Jones D. N. Barnes J. C. Kirkby D. L. Higgins G. A. (1995). Age-associated impairments in a test of attention: evidence for involvement of cholinergic systems. J. Neurosci. 15, 7282–7292. 7472482 10.1523/JNEUROSCI.15-11-07282.1995 PMC6578042

[B31] Kauer J. A. Malenka R. C. Nicoll R. A. (1988). NMDA application potentiates synaptic transmission in the hippocampus. Nature 334, 250–252. 10.1038/334250a0 2840582

[B32] Kemp A. Manahan-Vaughan D. (2004). Hippocampal long-term depression and long-term potentiation encode different aspects of novelty acquisition. Proc. Natl. Acad. Sci. U S A 101, 8192–8197. 10.1073/pnas.0402650101 15150407 PMC419579

[B33] Kemp A. Manahan-Vaughan D. (2008a). β-adrenoreceptors comprise a critical element in learning-facilitated long-term plasticity. Cereb. Cortex 18, 1326–1334. 10.1093/cercor/bhm164 17906333

[B34] Kemp A. Manahan-Vaughan D. (2008b). The hippocampal CA1 region and dentate gyrus differentiate between environmental and spatial feature encoding through long-term depression. Cereb. Cortex 18, 968–977. 10.1093/cercor/bhm136 17702951

[B35] Kitchigina V. Vankov A. Harley C. Sara S. J. (1997). Novelty-elicited, noradrenaline-dependent enhancement of excitability in the dentate gyrus. Eur. J. Neurosci. 9, 41–47. 10.1111/j.1460-9568.1997.tb01351.x 9042567

[B36] Kulla A. Manahan-Vaughan D. (2000). Depotentiation in the dentate gyrus of freely moving rats is modulated by D1/D5 dopamine receptors. Cereb. Cortex 10, 614–620. 10.1093/cercor/10.6.614 10859139

[B37] Kusuki T. Imahori Y. Ueda S. Inokuchi K. (1997). Dopaminergic modulation of LTP induction in the dentate gyrus of intact brain. Neuroreport 8, 2037–2040. 10.1097/00001756-199705260-00046 9223098

[B38] Larrabee G. J. McEntee W. J. (1995). Age-associated memory impairment: sorting out the controversies. Neurology 45, 611–614. 10.1212/wnl.45.4.611 7723944

[B40] Lemon N. Aydin-Abidin S. Funke K. Manahan-Vaughan D. (2009). Locus coeruleus activation facilitates memory encoding and induces hippocampal LTD that depends on β-adrenergic receptor activation. Cereb. Cortex 19, 2827–2837. 10.1093/cercor/bhp065 19435710 PMC2774396

[B39] Lemon N. Manahan-Vaughan D. (2006). Dopamine D1/D5 receptors gate the acquisition of novel information through hippocampal long-term potentiation and long-term depression. J. Neurosci. 26, 7723–7729. 10.1523/JNEUROSCI.1454-06.2006 16855100 PMC6674280

[B101] Lemon N. Manahan-Vaughan D. (2012). Dopamine D1/D5 receptors contribute to de novo hippocampal LTD mediated by novel spatial exploration or locus coeruleus activity. Cereb. Cortex. 22, 2131–2138. 10.1093/cercor/bhr297 22038910 PMC3412443

[B41] Li S. Cullen W. K. Anwyl R. Rowan M. J. (2003). Dopamine-dependent facilitation of LTP induction in hippocampal CA1 by exposure to spatial novelty. Nat. Neurosci. 6, 526–531. 10.1038/nn1049 12704392

[B42] Luine V. Bowling D. Hearns M. (1990). Spatial memory deficits in aged rats: contributions of monoaminergic systems. Brain Res. 537, 271–278. 10.1016/0006-8993(90)90368-l 2085779

[B100] Manahan-Vaughan D. (1997). Group 1 and 2 metabotropic glutamate receptors play differential roles in hippocampal long-term depression and long-term potentiation in freely moving rats. J. Neurosci. 17, 3303–3311. 9096163 10.1523/JNEUROSCI.17-09-03303.1997 PMC6573649

[B43] Manahan-Vaughan D. (2000). Long-term depression in freely moving rats is dependent upon strain variation, induction protocol and behavioral state. Cereb. Cortex 10, 482–487. 10.1093/cercor/10.5.482 10847598

[B44] Manahan-Vaughan D. Braunewell K.-H. Reymann K. H. (1998). Subtype-specific involvement of metabotropic glutamate receptors in two forms of long-term potentiation in the dentate gyrus of freely moving rats. Neuroscience 86, 709–721. 10.1016/s0306-4522(98)00111-0 9692711

[B45] Martin S. J. Grimwood P. D. Morris R. G. (2000). Synaptic plasticity and memory: an evaluation of the hypothesis. Annu. Rev. Neurosci 23, 649–711. 10.1146/annurev.neuro.23.1.649 10845078

[B46] Mishra J. de Villers-Sidani E. Merzenich M. Gazzaley A. (2014). Adaptive training diminishes distractibility in aging across species. Neuron 84, 1091–1103. 10.1016/j.neuron.2014.10.034 25467987 PMC4264379

[B47] Monfort P. Felipo V. (2007). Hippocampal long-term potentiation is reduced in mature compared to young male rats but not in female rats. Neuroscience 146, 504–508. 10.1016/j.neuroscience.2007.02.008 17395392

[B48] Neugebauer F. Korz V. Frey J. U. (2009). Modulation of extracellular monoamine transmitter concentrations in the hippocampus after weak and strong tetanization of the perforant path in freely moving rats. Brain Res. 1273, 29–38. 10.1016/j.brainres.2009.03.055 19345680

[B49] Ng G. Y. Mouillac B. George S. R. Caron M. Dennis M. Bouvier M.. (1994). Desensitization, phosphorylation and palmitoylation of the human dopamine D1 receptor. Eur. J. Pharmacol. 267, 7–19. 10.1016/0922-4106(94)90219-4 7515822

[B50] Ornstein K. Milon H. McRae-Degueurce A. Alvarez C. Berger B. Wiirzner H. P. (1987). Biochemical and radioautographic evidence for dopaminergic afferents of the locus coeruleus originating in the ventral tegmental area. J. Neural Transm. 70, 183–191. 10.1007/bf01253597 2445911

[B51] Orr G. Rao G. Houston F. P. McNaughton B. L. Barnes C. A. (2001). Hippocampal synaptic plasticity is modulated by theta rhythm in the fascia dentata of adult and aged freely behaving rats. Hippocampus 11, 647–654. 10.1002/hipo.1079 11811658

[B52] Paladini C. A. Roeper J. (2014). Generating bursts (and pauses) in the dopamine midbrain neurons. Neuroscience 282, 109–121. 10.1016/j.neuroscience.2014.07.032 25073045

[B53] Perlmutter M. Metzger R. Nezworski T. Miller K. (1981). Spatial and temporal memory in 20 to 60 year olds. J. Gerontol. 36, 59–65. 10.1093/geronj/36.1.59 7451838

[B54] Popova J. S. Petkov V. D. (1989). Age-related changes in rat brain muscarinic receptors and β-adrenoreceptors. Gen. Pharmacol. 20, 581–584. 10.1016/0306-3623(89)90089-x 2558040

[B55] Pöschel B. Manahan-Vaughan D. (2007). Persistent (<24 h) long-term depression in the dentate gyrus of freely moving rats is not dependent on activation of NMDA receptors, L-type voltage-gated calcium channels or protein synthesis. Neuropharmacology 52, 46–54. 10.1016/j.neuropharm.2006.07.019 16899259

[B56] Reis G. F. Lee M. B. Huang A. S. Parfitt K. D. (2005). Adenylate cyclase-mediated forms of neuronal plasticity in hippocampal area CA1 are reduced with aging. J. Neurophysiol. 93, 3381–3389. 10.1152/jn.00827.2003 15911893

[B58] Sara S. J. (2015). Locus coeruleus in time with the making of memories. Curr. Opin. Neurobiol. 35, 87–94. 10.1016/j.conb.2015.07.004 26241632

[B57] Sara S. J. Vankov A. Hervé A. (1994). Locus coeruleus-evoked responses in behaving rats: a clue to the role of noradrenaline in memory. Brain Res. Bull. 35, 457–465. 10.1016/0361-9230(94)90159-7 7859103

[B59] Schuligoi R. Fernandez J. Heavens R. P. Sirinathsinghji D. J. S. (1993). Decreased tyrosine hydroxylase mRNA but not cholecystokinin mRNA in the pars compacta of the substantia nigra and ventral tegmental area of aged rats. Brain Res. Mol. Brain Res. 19, 333–338. 10.1016/0169-328x(93)90135-c 7901729

[B60] Schultz W. (1998). Predictive reward signal of dopamine neurons. J. Neurophysiol. 80, 1–27. 9658025 10.1152/jn.1998.80.1.1

[B61] Schultz W. Apicella P. Ljungberg T. (1993). Responses of monkey dopamine neurons to reward and conditioned stimuli during successive steps of learning a delayed response task. J. Neurosci. 13, 900–913. 8441015 10.1523/JNEUROSCI.13-03-00900.1993 PMC6576600

[B62] Shirokawa T. Ishida Y. Isobe K.-I. (2000). Age-dependent changes in axonal branching of single locus coeruleus neurons projecting to two different terminal fields. J. Neurophysiol. 84, 1120–1122. 10.1016/s0168-0102(00)81585-0 10938337

[B63] Shukitt-Hale B. Casadesus G. Cantuti-Castelvetri I. Joseph J. A. (2001). Effect of age on object exploration, habituation and response to spatial and nonspatial change. Behav. Neurosci. 115, 1059–1064. 10.1037/0735-7044.115.5.1059 11584918

[B64] Siddiqi Z. Kemper T. L. Killiany R. (1999). Age-related neuronal loss from the substantia nigra-pars compacta and central tegmental area of the rhesus monkey. J. Neuropathol. Exp. Neurol. 58, 959–971. 10.1097/00005072-199909000-00006 10499438

[B65] Sierra-Mercado D. Dieguez D. Jr. Barea-Rodriguez E. J. (2008). Brief novelty exposure facilitates dentate gyrus LTP in aged rats. Hippocampus 18, 835–843. 10.1002/hipo.20447 18481283 PMC2910321

[B66] Simon H. Le Moal M. Stinus L. Calas A. (1979). Anatomical relationships between the ventral mesencephalic tegmentum—A 10 region and the locus coeruleus as demonstrated by anterograde and retrograde tracing techniques. J. Neural Transm. 44, 77–86. 10.1007/bf01252703 220380

[B67] Stanton P. K. Sarvey J. M. (1985). Depletion of norepinephrine, but not serotonin, reduces long-term potentiation in the dentate gyrus of rat hippocampal slices. J. Neurosci. 5, 2169–2176. 4040556 10.1523/JNEUROSCI.05-08-02169.1985 PMC6565305

[B68] Straube T. Frey J. U. (2003). Involvement of β-adrenergic receptors in protein synthesis-dependent late long-term potentiation (LTP) in the dentate gyrus of freely moving rats: the critical role of the LTP induction strength. Neuroscience 119, 473–479. 10.1016/s0306-4522(03)00151-9 12770561

[B69] Straube T. Korz V. Balschun D. Frey J. U. (2003). Requirement of β-adrenergic receptor activation and protein synthesis for LTP-reinforcement by novelty in rat dentate gyrus. J. Physiol. 552, 953–960. 10.1113/jphysiol.2003.049452 12937286 PMC2343450

[B70] Suzuki M. Hatano K. Sakiyama Y. Kawasumi Y. Kato T. Ito K. (2001). Age-related changes of dopamine D1-like and D2-like receptor binding in the F344/N rat striatum revealed by positron emission tomography and *in vitro* receptor autoradiography. Synapse 41, 285–293. 10.1002/syn.1085 11494399

[B71] Szot P. Franklin A. Sikkema C. Wilkinson C. W. Raskind M. A. (2012). Sequential loss of LC noradrenergic and dopaminergic neurons results in a correlation of dopaminergic neuronal number to striatal dopamine concentration. Front. Pharmacol. 3:184. 10.3389/fphar.2012.00184 23129999 PMC3487487

[B72] Tenorio G. Connor S. A. Guévremont D. Abraham W. C. Williams J. O’Dell T. J.. (2010). “Silent” priming of translation-dependent LTP by β-adrenergic receptors involves phosphorylation and recruitment of AMPA receptors. Learn. Mem. 17, 627–638. 10.1101/lm.1974510 21097606 PMC2998333

[B73] Twarkowski H. Hagena H. Manahan-Vaughan D. (2016). The 5-Hydroxytryptamine4 (5-HT4) receptor enables differentiation of informational content and encoding in the hippocampus. Hippocampus 26, 875–891. 10.1002/hipo.22569 26800645 PMC5067691

[B74] Ungless M. A. (2004). Dopamine: the salient issue. Trends Neurosci. 27, 702–706. 10.1016/j.tins.2004.10.001 15541509

[B76] Wiescholleck V. André M. A. Manahan-Vaughan D. (2014). Early age-dependent impairments of context-dependent extinction learning, object recognition and object-place learning occur in rats. Hippocampus 24, 270–279. 10.1002/hipo.22220 24132946

[B75] Wiescholleck V. Manahan-Vaughan D. (2014). Antagonism of D1/D5 receptors prevents long-term depression (LTD) and learning-facilitated LTD at the perforant path-dentate gyrus synapse in freely behaving rats. Hippocampus 24, 1615–1622. 10.1002/hipo.22340 25112177

